# Sample Size Recalculation in Adaptive Group Sequential Study Designs for Comparing Restricted Mean Survival Times

**DOI:** 10.1002/sim.70490

**Published:** 2026-03-18

**Authors:** Carolin Herrmann, Paul Blanche

**Affiliations:** ^1^ Mathematical Institute Heinrich Heine University Düsseldorf Düsseldorf Germany; ^2^ Center for Digital Medicine Heinrich Heine University Düsseldorf Düsseldorf Germany; ^3^ Section of Biostatistics University of Copenhagen Copenhagen K Denmark

**Keywords:** adaptive clinical trial, delayed treatment effect, non‐proportional hazards, restricted mean survival time, sample size adaptation, time‐to‐event analysis

## Abstract

Non‐proportional hazards cases are frequently expected in clinical trials with time‐to‐event endpoints (e.g., cardiology, oncology). The relevance of hazard ratios to quantify the treatment effect is questionable and potentially misleading in this context. Hence, alternative methods comparing restricted mean survival times are increasingly promoted. Specific challenges arise when planning clinical trials for comparing restricted mean survival times, as several nuisance parameter estimates are needed for calculating the sample size. Precise estimates might be difficult to obtain at the planning stage and might lead to underpowered trials. One way of dealing with this insecurity is to apply adaptive group sequential study designs with the option to adapt the sample size during an ongoing trial. Within this work, we consider such sample size adaptations, with a specific focus on the context of delayed treatment effects. We compare the performance of an adaptive design with the restricted mean survival time as the primary endpoint with other commonly chosen endpoints in this scenario by means of an extensive simulation study. With our proposed method, adaptive designs with the restricted mean survival time as the primary endpoint are now thoroughly explained. The combination test that we describe can also be useful for other adaptations than sample sizes.

## 1 Introduction

The time until a specific type of event is often of primary interest in clinical trials. The log‐rank test and Cox proportional hazards models [1] are well established, and extended research has been conducted. When it comes to non‐proportional hazards, challenges arise with regard to the choice of the method to apply. Different alternative effect measures have been proposed (e.g., restricted mean survival time [2, 3], t‐year survival, average hazard ratio [4, 5], MaxCombo test [6, 7]), but all approaches have their advantages and disadvantages. Non‐proportional hazards, however, are faced in many different clinical trials, for example, in cardiology and oncology, when a delayed treatment effect is expected. Irrespective of the endpoint type, another challenge one has to deal with when planning a clinical trial is to address potential insecurities about underlying parameter estimates needed for sample size calculation (e.g., event and dropout rates). One way of addressing this insecurity about the underlying parameters is to apply group sequential trial designs or even adaptive (group sequential) designs. While group sequential trials provide the option to stop a trial early for efficacy or futility, adaptive group sequential designs offer even more flexibility by adapting parts of the study design, for example, adapting the sample size at an interim analysis based on which effect size and/or nuisance parameters have been observed at that time point. Time‐to‐event trials usually take a long time, so here it seems especially favorable to add interim looks. Methods for group sequential and adaptive designs are well established for continuous, binary, and time‐to‐event endpoints with proportional hazards, see [8] for an overview. For designs with a possible sample size update under proportional hazards, some methods have been proposed [9, 10, 11, 12]. However, under non‐proportional hazards, the developed methods are a lot more sparse. Our research was motivated by a case in cardiology that we met during a statistical consultation, about the planning of a clinical trial for which a delayed treatment effect was expected. The restricted mean survival time was considered as an interesting endpoint in that example, especially because the follow‐up time was planned to be the same for all patients (τ= 1.5 years). For details, we refer the interested reader already to the clinical data example presented in Section 4. Our clinical example was less motivated by the fact to stop the trial early (where corresponding group sequential trials had already been developed for the restricted mean survival time [13]) but more by having the option to adapt the sample size. We refer to [14] for an interesting discussion on the advantages and disadvantages of adaptive trial designs with sample size recalculation compared to “usual” group sequential designs. Our goal was to specify a trial design in which sample size recalculation with the restricted mean survival time as primary endpoint is possible, motivated by the cardiologic example with expecting non‐proportional hazards and having the above mentioned natural cutoff at τ years. Another similar application scenario can be found in Reference [15] on the topic of children's oncology. Here, the t‐year survival was chosen as estimand instead of the RMST, see also [14]. They considered the conditional power for survival at t years. For a discussion of different estimands, we refer the interested reader to [16], and general discussions on the RMST can be found, for example, in References [17, 18].

The goal of this article is to present a methodology to design and analyze an adaptive clinical trial with sample size recalculation at interim analysis, when the restricted mean survival time is the primary endpoint. Especially, we focus on the situation of non‐proportional hazards with a delayed treatment effect. Our aim is to provide trialists with new options to consider, to make better design choices, or to take better‐informed decisions about design choices. Despite the fact that the log‐rank test might still be more powerful than a test comparing RMSTs in this situation, the estimand and comparison associated with this test are not well defined in this situation. By contrast, the restricted mean survival time (RMST) is an interesting estimand under non‐proportional hazards [19]. The structure of the article is as follows: In Section 2, we present the methodology with notations, the adaptive group sequential testing framework, the sample size adaptation procedure, and important details about trial planning. Afterwards, Section 3 presents an extensive simulation study, in which we compare the methodology with some alternatives. We provide further details about our motivating example from cardiology in Section 4. We conclude with a discussion in Section 5.

## 2 Notation and Methods

### 2.1 Restricted Mean Survival Time

For addressing the non‐proportional hazards setting, the difference in restricted mean survival time (RMST) is used to define the treatment effect. The RMST describes the average survival from time t=0 to a pre‐specified time point τ (e.g., 2 years). In other words, the average number of days or years alive within the τ days or years after treatment initiation. It is therefore the expectation of a truncated survival time $X_{\tau }=\min(T,\tau )$, denoted by $\mu_{\tau }$, and it can be written as

$$\begin{array}{l} \mu_{\tau }=\int_{0}^{\tau }S(t)dt, \end{array}$$

with survival function S(t)=ℙ(T>t) and random variable T (e.g., time to death) as described in Reference [17].

In trials comparing the survival functions of an intervention group I and a control group C, the difference in restricted mean survival time can be written as

(1) $$\begin{array}{l} \Delta =\mu_{\tau }^{\left(I\right)}-\mu_{\tau }^{\left(C\right)}=\int_{0}^{\tau }\left(S^{\left(I\right)}(t)-S^{\left(C\right)}(t)\right)dt. \end{array}$$

Hence, Δ describes the area between the two survival curves up to time τ. We are interested in a one‐sided test problem with the null hypothesis $H_{0}:\Delta <0$ versus the alternative hypothesis $H_{1}:\Delta \geq 0$.

To estimate Δ, the Kaplan–Meier estimator ${\hat{S}}^{\left(i\right)}(\cdot )$ for the survival function per group i∈{I,C} can be used, that is,

(2) $$\begin{array}{l} \hat{\Delta }=\int_{0}^{\tau }\left({\hat{S}}^{\left(I\right)}(t)-{\hat{S}}^{\left(C\right)}(t)\right)dt. \end{array}$$

This method handles censored data due to loss of follow‐up within τ years, or the fact that some patients can be followed for less than τ years at the time of interim analysis. The Kaplan–Meier estimator also handles data that are left truncated on top of being right censored (see, e.g., [20]). We will see why this matters for adaptive designs in Section 2.2. Left truncation is rarely encountered in randomized clinical trials, unlike in epidemiology.

### 2.2 The RMST in the Adaptive Group Sequential Testing Setting

Recently, Lu and Tian [13], building on Murray and Tsiatis [21], considered the restricted mean survival time in a classic group sequential setting for an arbitrary number of stages. Within this work, we restrict ourselves to the two‐stage setting, which means that we have one interim analysis based on which the trial might be stopped early, and otherwise it continues with the second stage. In case of continuation, we allow adaptations to the sample size. Hence, we are in the adaptive group sequential setting.

#### 2.2.1 General Framework

The interim analysis takes place $t_{int}$ years after the start of accrual. Unless the trial stops prematurely, accrual of patients continues after the interim analysis, and the final analysis is conducted at the time $t_{final}$. Both for the interim analysis and the final analysis, the same truncation time to define the RMST is chosen, that is, $\tau_{int}=\tau_{final}=\tau$. Hence, the same estimand is considered at the interim and final analysis. Except for losses to follow up, we assume that every patient is exactly observed for the duration τ, which also applies to the lastly recruited patient. This is similar to, for example, Schmidli et al. [15].

Due to the adaptive “two‐stage” feature of the trial design, which enables sample size recalculation, we consider a combination test for analyzing the data at the end of the trial [22]. Hence, we split the patients' data into two parts as often done [8]. Let therefore $E_{j}$ describe the calendar time of enrollment per patient, $T_{j}$ the time from entering the trial to the event of interest (e.g., death), and $C_{j}$ the time from entering the trial to (potential) dropout. All $E_{j},T_{j}$ and $C_{j}$ are assumed to be mutually independent.

The *first‐stage data*, denoted by $X_{1}$, consist of data from individuals who were recruited before the interim analysis and followed up until $t_{int}$. Formally,

$$\begin{array}{l} X_{1}=\{({\tilde{T}}_{j}^{\left(1\right)},\delta_{j}^{\left(1\right)},A_{j}),i=1,\ldots ,n_{1}\}, \end{array}$$

where ${\tilde{T}}_{j}^{\left(1\right)}=\min({\tilde{T}}_{j},L_{j})$ and $\delta_{j}^{\left(1\right)}=\delta_{j}1({\tilde{T}}_{j}\leq L_{j})$ are the censored time observed and the corresponding (non‐)censoring indicator, while ${\tilde{T}}_{j}=\min(T_{j},C_{j},\tau )$ and $\delta_{j}=1(T_{j}\leq \min(C_{j},\tau ))$ are those observed at the final analysis, and $L_{j}=t_{int}-E_{j}$ is the time from enrollment to interim analysis. Equivalently, $\delta_{j}^{\left(1\right)}$ can be written as $1\{T_{j}\leq \min(C_{j},\tau ,t_{int}-E_{j})\}$. In addition, $A_{j}$ denotes the treatment group (I for intervention, C for control) and $n_{1}$ denotes the number of patients accrued before the interim analysis.

**FIGURE 1:**
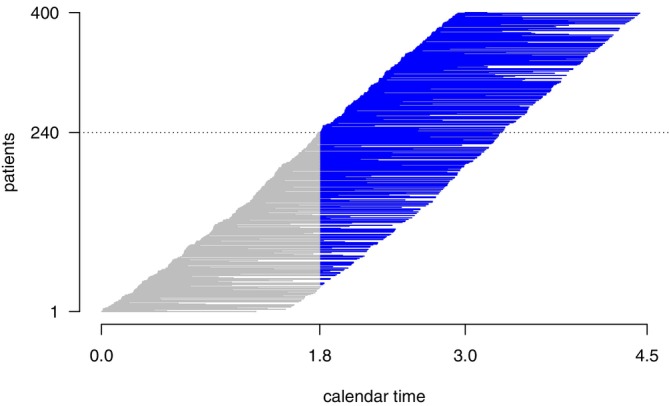
Example of data split into right‐censored data $X_{1}$ (gray) contributing to the first stage and left‐truncated data $X_{2}$ (blue) contributing to the second stage. Each horizontal segment represents the time from the date of inclusion into the trial until the date of the event or loss of follow‐up. Blue segments represent follow up data of patients included in stage 1, that is, $X_{2}^{\left(1\right)}$ (until patient number 240) and from newly recruited patients in the second stage $X_{2}^{\left(2\right)}$ (from patient number 241). Here, $t_{int}=2.0$ and τ=1.5. Patients are accrued for 3 years, leading to $t_{final}=t_{int}+\tau =4.5$years. The first stage sample size is given by $n_{1}=400\cdot 1.8/(3.0-0.0)=240$.

The *second‐stage data*, denoted by $X_{2}$, is composed of two parts. One part, denoted by $X_{2}^{\left(1\right)}$, consists of further follow‐up data from patients who entered the trial before the interim analysis, but for whom further follow‐up data were collected after the interim analysis. These patients are those who entered the trial less than τ years before the interim analysis, for whom we have not observed an event or a dropout until the interim analysis. The second part of the data, denoted by $X_{2}^{\left(2\right)}$, comes from patients who entered the trial after the interim analysis. Formally,

$$\begin{array}{ll} X_{2} & =X_{2}^{\left(1\right)}\cup X_{2}^{\left(2\right)}\\ & =\{({\tilde{T}}_{j},\delta_{j},L_{j},A_{j}),j=n_{1}-{\bar{n}}_{1}+1,\ldots ,n_{1}+n_{2}\}, \end{array}$$

where $X_{2}^{\left(1\right)}$ and $X_{2}^{\left(2\right)}$ denote the data in $X_{2}$ coming from “stage 1 patients” $j=n_{1}-{\bar{n}}_{1}+1,\ldots ,n_{1}$, included before interim, and “stage 2 patients” $j=n_{1}+1,\ldots ,n_{1}+n_{2}$, included after interim, respectively. Here, ${\bar{n}}_{1}=n_{1}-\sum_{j=1}^{n_{1}}1({\tilde{T}}_{j}^{\left(1\right)}<{\tilde{T}}_{j})$ denotes the (random) number of stage 1 patients of whom further follow‐up data are collected after interim analysis. We set $L_{j}=0$ to all “stage 2 patients”, that is $L_{j}=\max(t_{int}-E_{j},0)$ for all j. Note that, unlike stage 1 data, stage 2 data are left‐truncated on top of being right‐censored, and $L_{j}$ is the truncation time. The left truncation comes from the fact that stage 1 patients are included in the stage 2 data only if they are event‐free at the time of interim analysis. A graphical representation of stage 1 and stage 2 data is given in Figure 1.

The idea of splitting the data from patients included before the interim analysis into $X_{1}$ and $X_{2}^{\left(1\right)}$ for making inference goes back to Keiding, Bayer and Watt‐Boolsen [23] and has been considered in detail by Jahn‐Eimermacher and Ingel [24]. They showed that the stage‐wise data $X_{1}$ and $X_{2}$ can be analyzed as independent and therefore are suitable for a combination test.

#### 2.2.2 Stage‐Wise Testing Strategy

The restricted mean survival time estimator based on the first‐stage data is denoted by $\hat{\Delta }(X_{1})$ and for the second‐stage data by $\hat{\Delta }(X_{2})$. Asymptotically, that is as $n_{1}\to \infty$, we have $\sqrt{n_{1}}\{\hat{\Delta }(X_{1})-\Delta \}∼N(0,\sigma_{1}^{2})$, for some $\sigma_{1}>0$. Similarly, $\sqrt{n_{2}+{\bar{n}}_{1}}\{\hat{\Delta }(X_{2})-\Delta \}∼N(0,\sigma_{2}^{2})$, for some $\sigma_{2}>0$, as $n_{2}\to \infty$ and ${\bar{n}}_{1}\to \infty$ [25]. Consistent estimators of $\sigma_{1}$ and $\sigma_{2}$ can therefore be obtained by

$$\begin{array}{ll} {\hat{\sigma }}_{1}^{2} & =n_{1}/2\left[{\left\{{\hat{\sigma }}_{{\hat{\mu }}_{\tau }}^{\left[I\right]}(X_{1})\right\}}^{2}+{\left\{{\hat{\sigma }}_{{\hat{\mu }}_{\tau }}^{\left([C\right]}(X_{1})\right\}}^{2}\right],\\ {\hat{\sigma }}_{2}^{2} & =(n_{2}+{\bar{n}}_{1})/2\left[{\left\{{\hat{\sigma }}_{{\hat{\mu }}_{\tau }}^{\left[I\right]}(X_{2})\right\}}^{2}+{\left\{{\hat{\sigma }}_{{\hat{\mu }}_{\tau }}^{\left[C\right]}(X_{2})\right\}}^{2}\right], \end{array}$$

with ${\hat{\sigma }}_{{\hat{\mu }}_{\tau }}^{\left[i\right]}(X_{k})$ being the standard error of the RMST estimator ${\hat{\mu }}_{\tau }^{\left[i\right]}(X_{k})$, compare Appendix A for details.

We define the following stage‐wise testing strategy. We reject the null hypothesis after the first stage if $Z_{1}\geq c_{1}$, where $Z_{1}=\sqrt{n_{1}}\hat{\Delta }(X_{1})/{\hat{\sigma }}_{1}$, for some $c_{1}>0$ defined below. The null hypothesis is rejected after the final stage if the test statistic based on the inverse normal combination test (cf. [22, 26])

(3) $$\begin{array}{l} Z_{final}=w_{1}\cdot Z_{1}+w_{2}\cdot Z_{2} \end{array}$$

is greater or equal to $c_{final}$, that is, $Z_{final}\geq c_{final}$, with pre‐specified weights $w_{1}$ and $w_{2}$ such that $w_{1}^{2}+w_{2}^{2}=1$. The choice of the weights will be discussed in Section 2.4. Here, $c_{1}$ and $c_{final}$ denote the critical values after the first stage and at the final analysis, respectively, and $Z_{2}=\sqrt{n_{2}+{\bar{n}}_{1}}\hat{\Delta }(X_{2})/{\hat{\sigma }}_{2}$.

To define $c_{1}$ and $c_{final}$, we suggest to use the alpha‐spending function approach [27], which is common in group sequential designs with survival data [28], as the information level at the interim and final analysis is difficult to anticipate precisely at the planning stage of the trial. To define the critical value $c_{1}$ in the rest of this manuscript, we will use the O'Brien–Fleming type alpha‐spending function [29] $\tilde{\alpha }({\hat{I}}_{i})=2\cdot \{1-\Phi [\Phi^{-1}(1-\alpha /2)/\sqrt{{\hat{I}}_{i}/I_{max}}]\}$, where ${\hat{I}}_{i}$ is the observed information at stage i=1,2. Also, $I_{max}$ denotes the planned maximum information level and α is the global one‐sided significance level. The information level at the interim analysis is ${\hat{I}}_{1}=n_{1}/{\hat{\sigma }}_{1}^{2}$, at the final analysis it is ${\hat{I}}_{final}={\hat{I}}_{1}+{\hat{I}}_{2}$ with ${\hat{I}}_{2}=(n_{2}+{\bar{n}}_{1})/{\hat{\sigma }}_{2}^{2}$ and the (planned) maximum information level is $I_{max}={\left\{\Phi^{-1}(\alpha )+\Phi^{-1}(\beta )\right\}}^{2}/\Delta_{0}^{2}\cdot \gamma$, where 1−β is the power planned for under the alternative hypothesis $\Delta =\Delta_{0}$ and γ the inflation factor, see, for example, [30] or [31]. For the O'Brien–Fleming adjustment, γ is approximately 1.01 with the 1:1 allocation ratio considered in this manuscript, see, for example, [30].

The local rejection boundary $c_{1}$, for the standardized test statistic $Z_{1}$, is defined by solving

$$\begin{array}{l} {\mathbb{P}}_{H_{0}}(Z_{1}\geq c_{1})=\tilde{\alpha }({\hat{I}}_{1}), \end{array}$$

that is, $c_{1}=\Phi^{-1}(1-\tilde{\alpha }({\hat{I}}_{1}))$. In the notation ${\mathbb{P}}_{H_{0}}$, the subscript $H_{0}$ emphasizes that we compute the probability under the null hypothesis Δ=0, where (asymptotically) $Z_{1}∼N(0,1)$. Similarly, we define $c_{final}$ by solving

$$\begin{array}{ll} {\mathbb{P}}_{H_{0}}(Z_{final}\geq c_{final},Z_{1}<c_{1}) & =\alpha -\tilde{\alpha }({\hat{I}}_{1}), \end{array}$$

and using the fact that under the null hypothesis (asymptotically)

$$\begin{array}{l} \left(\begin{array}{l} Z_{1}\\ Z_{final} \end{array}\right)∼N\left(\left(\begin{array}{c} 0\\ 0 \end{array}\right),\left(\begin{array}{cc} 1 & w_{1}\\ w_{1} & 1 \end{array}\right)\right). \end{array}$$

The covariance $w_{1}$ follows from $\text{Cov}(Z_{1},Z_{final})=w_{1}\text{Cov}(Z_{1},Z_{1})+w_{2}\text{Cov}(Z_{1},Z_{2})$ and $\text{Cov}(Z_{1},Z_{2})=0$; the latter being a consequence of the independent increment structure among the test statistic at interim and final analysis, in a group sequential trial comparing the restricted mean survival time via the areas under the Kaplan–Meier curve as described above [21]. The critical values $c_{1}$ and $c_{final}$ can only be calculated after the data at interim $X_{1}$ have been observed, as they depend on the observed level of information ${\hat{I}}_{1}$.

#### 2.2.3 Alternative Testing Strategies

Next to the truncated data approach as introduced above, we consider two alternative testing strategies, one based on Desseaux and Porcher [11], the other one is a combination of the truncated approach and the one by Desseaux and Porcher. First of all, let $X_{all}=\{({\tilde{T}}_{j},\delta_{j},A_{j}),j=1,...,n\}$ denote the fully observed data at the end of the trial for *all* patients with $n=n_{1}+n_{2}$. Using these data $X_{all}$, one can also compute the corresponding estimators $\hat{\Delta }(X_{all})=\int_{0}^{\tau }\left({\hat{S}}_{X_{all}}^{\left[I\right]}(t)-{\hat{S}}_{X_{all}}^{\left[C\right]}(t)\right)dt$ and

$$\begin{array}{ll} {\hat{\sigma }}_{all}^{2} & =(n_{1}+n_{2})/2\left[{\left\{{\hat{\sigma }}_{{\hat{\mu }}_{\tau }}^{\left[I\right]}(X_{all})\right\}}^{2}+{\left\{{\hat{\sigma }}_{{\hat{\mu }}_{\tau }}^{\left[C\right]}(X_{all})\right\}}^{2}\right]. \end{array}$$

The corresponding test statistic is $Z_{all}=\sqrt{n_{1}+n_{2}}\hat{\Delta }(X_{all})/{\hat{\sigma }}_{all}=\hat{\Delta }(X_{all})\sqrt{{\hat{I}}_{all}}$, where ${\hat{I}}_{all}=n/{\hat{\sigma }}_{all}^{2}$ denotes the observed information obtained from data $X_{all}$.

The first alternative final test statistic, following Desseaux and Porcher [11], is defined by

(4) $$\begin{array}{l} Z_{final}^{\prime }=w_{1}\cdot Z_{1}+w_{2}\cdot \left[\frac{Z_{all}\sqrt{{\hat{I}}_{all}}-Z_{1}\sqrt{{\hat{I}}_{1}}}{\sqrt{{\hat{I}}_{all}-{\hat{I}}_{1}}}\right]. \end{array}$$

In short, $Z_{2}$ in the definition of $Z_{final}$ in Equation (3) is replaced by

$$Z_{2}^{\prime }=\left\{Z_{all}\sqrt{{\hat{I}}_{all}}-Z_{1}\sqrt{{\hat{I}}_{1}}\right\}/\left\{\sqrt{{\hat{I}}_{all}-{\hat{I}}_{1}}\right\}$$

in the definition of $Z_{final}^{\prime }$ because $Z_{2}$ and $Z_{2}^{\prime }$ are asymptotically equivalent. See, for example, Section 3 in Reference [31] for standard properties of the canonical joint distribution and [21] to justify that it holds asymptotically for $\left(Z_{1},Z_{all}\right)$. An incentive to consider $Z_{2}^{\prime }$ instead of $Z_{2}$ was the suspicion that small sample performances of methods using truncated data might be unsatisfactory.

The second alternative that we consider is a midway approach between the truncated approach with $Z_{2}$ and the Desseaux–Porcher approach with $Z_{2}^{\prime }$. We define the second alternative by replacing $Z_{2}=\hat{\Delta }(X_{2})\sqrt{{\hat{I}}_{2}}$ in the definition of $Z_{final}$ by $Z_{2}^{\prime \prime }=\hat{\Delta }(X_{2})\sqrt{{\hat{I}}_{all}-{\hat{I}}_{1}}$. That is,

(5) $$\begin{array}{l} Z_{final}^{\prime \prime }=w_{1}\cdot Z_{1}+w_{2}\cdot \hat{\Delta }(X_{2})\sqrt{{\hat{I}}_{all}-{\hat{I}}_{1}}. \end{array}$$

The rationale for this test statistic also comes from the results from Reference [21], which imply that ${\hat{I}}_{all}={\hat{I}}_{1}+{\hat{I}}_{2}+o_{p}(n)$.

The consequent decisions on rejecting the null hypothesis or not are then again based on the same α‐spending approach and critical value $c_{final}$ as described above.

### 2.3 Conditional Power and Sample Size Adaptations

Due to planning uncertainties regarding the sample size, it seems appealing to consider sample size adaptations at the interim analysis. The underlying idea is to make the sample size adaptations depend on the chance of a potential trial success if the trial continues the way it is (usually with a pre‐specified maximally feasible upper limit of the sample size, denoted by $n_{max}$). This refers to the well‐known concept of updating the sample size based on the conditional power. The conditional power is the probability of correctly rejecting the null hypothesis at the end of the trial given the test statistic observed at interim analysis ($Z_{1}$) and our best guesses of the values of the nuisance parameters, which are essential for power calculation (e.g., event rates in both groups, dropout rate). The guesses can now be made more precisely using the interim data $X_{1}$. The conditional power is therefore given by ${\mathbb{P}}_{H_{1}}(Z_{final}\geq c_{final}\;|\;Z_{1}=z_{1})$, where the subscript $H_{1}$ emphasizes that we compute the probability under the alternative hypothesis $\Delta =\Delta_{0}$, for some $\Delta_{0}>0$. In our setting, it is given by

(6) $$\begin{array}{ll} CP(\Delta_{0},z_{1}) & =1-\Phi \left(\frac{c_{final}-w_{1}z_{1}}{w_{2}}-\Delta_{0}\sqrt{\frac{{\bar{n}}_{1}+n_{2}}{\sigma_{2}^{2}}}\right)\\ & =1-\Phi \left(\frac{c_{final}-w_{1}z_{1}}{w_{2}}-\Delta_{0}\sqrt{\frac{{\bar{n}}_{1}}{\sigma_{21}^{2}}+\frac{n_{2}}{\sigma_{1*}^{2}}}\right). \end{array}$$

The first equality follows because (asymptotically) $Z_{2}∼N(\sqrt{{\bar{n}}_{1}+n_{2}}\Delta_{0}/\sigma_{2},1)$ under the alternative hypothesis. The second is due to the (asymptotic) decomposition

(7) $$\frac{{\bar{n}}_{1}/(n_{2}+{\bar{n}}_{1})}{\sigma_{21}^{2}}+\frac{n_{2}/(n_{2}+{\bar{n}}_{1})}{\sigma_{1*}^{2}}=\frac{1}{\sigma_{2}^{2}}+o_{p}(1)$$

as ${\bar{n}}_{1}\to \infty$ and $n_{2}\to \infty$, which holds as a consequence of the independent increment structure among the test statistic at interim and final analysis, as shown by [21]. Essentially, Equation (7) states that the level of information coming from the second‐stage data $X_{2}=X_{2}^{\left(1\right)}\cup X_{2}^{\left(2\right)}$ is the sum of two information levels. The first comes from the additional follow‐up data of patients already included in stage 1, that is $X_{2}^{\left(1\right)}$, and the second comes from the newly included patients at stage 2, that is, $X_{2}^{\left(2\right)}$. Here, ${\bar{n}}_{1}/(n_{2}+{\bar{n}}_{1})$ and $n_{2}/(n_{2}+{\bar{n}}_{1})$ can be thought of as “weights” and represent the proportions of patients included before and after interim analysis among those who contribute to stage 2 data $X_{2}$, respectively. Also, note that Equation (7) uses the notation $\sigma_{1*}$, which denotes the limit of ${\hat{\sigma }}_{all}$ when n→∞. Moreover, we also use the conditional power to define a non‐binding futility stopping criterion, that is, when the conditional power, $CP(\Delta_{0},z_{1})$, based on the initially planned sample size for the second stage, falls below some pre‐defined conditional power boundary $CP_{min}$.

In those cases where the trial is not stopped early due to efficacy or futility, the stage 2 sample size $n_{2}$ can be chosen to account for what we observed and learned from the stage 1 data $X_{1}$. The idea underlying this sample size adaptation is to set $CP(\Delta_{0},z_{1})=1-\beta_{cond}$ and find the smallest sample size $n_{2}$ that assures a conditional power of $1-\beta_{cond}$, by solving Equation (6). To solve Equation (6) for $n_{2}$, we need to set values for $\sigma_{21}$ and $\sigma_{1*}$ which are ideally our “best guesses” based on the observed stage 1 data $X_{1}$. Of course, these best guesses should be compatible with the RMST difference $\Delta_{0}$ for which we want to power the study. Hence, careful thinking is needed because the two asymptotic standard deviations $\sigma_{21}$ and $\sigma_{1*}$ depend on the survival functions in each treatment group and obviously $\Delta_{0}$ too. In other words, there is therefore some variation dependence between the three parameters $\sigma_{21}$, $\sigma_{1*}$, and $\Delta_{0}$. A method to estimate relevant values for $\sigma_{1*}$ and $\sigma_{21}$ is presented in the Appendix. It consists of a simulation algorithm, for which important data‐generating parameters are estimated from the interim data using constrained maximum likelihood, as detailed in Appendix A.

#### 2.3.1 Simulating Data in the Delayed Treatment Effect Context

In this paper, we focus on the following specific context, which will be pivotal to simulate appropriate data (cf. Step 1(a) in the Appendix A). We aim to randomize patients 1:1, and we assume that (at least approximately) we can expect that the hazard of the time‐to‐event T in each arm is piece‐wise constant. Specifically, we assume that a “delayed treatment effect” is expected and that the hazard is expected to be (at least approximately) constant and identical in the two arms until a specific time $t_{0}$. Let us denote this hazard by $\lambda_{0}$. After time $t_{0}$, we assume that the hazards in the two arms are still (approximately) constant, but different between the two groups. We denote them by $\lambda_{I}$ and $\lambda_{C}$. Formally,

$$\begin{array}{ll} & \underset{dt\to 0}{\lim}\;\mathbb{P}(t\leq T<t+dt|T\geq t,A=i)/dt=\lambda_{0}\cdot 1(t\leq t_{0})\\ & \;+\lambda_{i}\cdot 1(t>t_{0})\;\text{for}\;i\in \{I,C\}. \end{array}$$

Note that the assumptions regarding the hazards do not need to hold for the non‐parametric inference detailed above to be valid in terms of type‐I error control. They only need to hold approximately to provide meaningful sample size and power calculations. Furthermore, note that considering this “delayed treatment effect” setting is not uncommon, see, for example, the simulation studies of [13, 16, 32].

We will further assume that, as often, patients are expected to be accrued uniformly. That is, the entry time E is expected to follow a uniform distribution in $\left[0,t_{E}\right]$. We further assume to expect some loss of follow‐up during the trial and that the time to loss‐of‐follow‐up follows (approximately) an exponential distribution with rate c. We will assume that all patients are planned to be followed up for τ years (e.g., [15]). Hence, in total, the trial is expected to last $t_{E}+\tau$ years, from first inclusion to last completion of the τ years follow‐up. For any values of rate parameters c, $\lambda_{0}$, $\lambda_{C}$ and $\lambda_{I}$, times $t_{0}$ and τ and sample size $n_{1}$, one can easily simulate a dataset that fulfills the above assumptions (cf. also details in Appendix A).

### 2.4 Trial Planning: Defining $n_{1}$ and Pre‐Specifying $w_{1}$ and $w_{2}$

To start the adaptive trial outlined above, one needs to first choose a relevant time τ, calculate a relevant first‐stage sample size $n_{1}$, and pre‐specify reasonable weights $w_{1}$ and $w_{2}$. This will typically depend on the choice of the time $t_{int}$ at which to perform the interim analysis. Once the RMST difference $\Delta_{0}$ for which we want to power the trial has been chosen—together with the desired power 1−β and the type‐I error α—it is possible to compute the (planned) maximum information level $I_{max}=\gamma \cdot {\left\{\Phi^{-1}(\alpha )+\Phi^{-1}(\beta )\right\}}^{2}/\Delta_{0}^{2}$. Accordingly, we should plan the trial with a sample size $n=n_{1}+n_{2}$ such that ${\hat{I}}_{all}=n/{\hat{\sigma }}_{all}^{2}\approx {\hat{I}}_{1}+{\hat{I}}_{2}$ is expected to be approximately equal to $I_{max}$. As ${\hat{\sigma }}_{all}$ converges towards $\sigma_{1*}$, we can therefore compute a first total sample size using the formula

(8) $$n=\gamma \cdot \frac{{\left\{\Phi^{-1}(\alpha )+\Phi^{-1}(\beta )\right\}}^{2}}{{\left(\Delta_{0}/\sigma_{1*}\right)}^{2}},$$

see also [17] for a similar derivation of this formula. As already discussed in Section 2.3.1 and details in Appendix A, based on initial guesses of rate parameters c, $\lambda_{0}$, $\lambda_{C}$ and the time $t_{0}$, one can compute the corresponding values for $\sigma_{1*}$ by simulations and then deduce the initial sample size n using Equation (8). For a given time of interim analysis $t_{int}$ and accrual duration $t_{E}$, one can therefore deduce $n_{1}$ as $n_{1}=n\cdot (t_{int}/t_{E})$ (assuming constant accrual rate).

The weights $w_{1}$ and $w_{2}$ should be chosen as the square root of the expected fraction of the maximal information observed at interim analysis, that is, $\sqrt{{\hat{I}}_{1}/I_{max}}$, to maximize the power of the combination test [8]. As ${\hat{I}}_{1}=n_{1}/{\hat{\sigma }}_{1}^{2}$, we can compute a numerical value for $\sigma_{1}$ by simulation as discussed in Section 2.3.1 and details in Appendix A, and then define $w_{1}=\sqrt{\left(n_{1}/\sigma_{1}^{2})/(n/\sigma_{1*}^{2}\right)}$ and $w_{2}=\sqrt{1-w_{1}^{2}}$.

Note that we implicitly assumed an accrual rate of $n/t_{E}$ patients per year. If this accrual rate is not achievable, another value of $t_{E}$ (and likely also of $t_{int}$) should be used, and $n_{1}$ and weights $w_{1}$ and $w_{2}$ should be recalculated accordingly.

## 3 Simulation Study

### 3.1 Simulation Framework

The evaluation goals of our simulation study can be stated as follows:

- Type‐I error control assessment,
- Power comparison with the log‐rank test and a test comparing survival probabilities at τ years,
- Studying the impact of the timing of the interim analysis,
- Illustration that the design can partly correct for wrong guesses during the initial planning.

Simulations were conducted with R version 4.4.2. We ran ten different Monte Carlo simulation studies (referred to as scenarios), each with $n_{MC}=10\;000$ runs and using L=100 for sample size adaptation at interim. For the one‐sided testing setting, we set α=0.025 and β=0.2. Local significance levels were retrieved according to an O'Brien–Fleming α‐spending approach [33]. In the sample size adaptations, a conditional power of 80% was set as the goal and the maximally feasible sample size $n_{max}$ was assumed to be $1.5\cdot n_{fix,guess}$, where $n_{fix,guess}$ is the sample size of a standard one‐stage clinical trial design calculated based on the initial guesses for the underlying parameter values. Accrual was assumed to take place within three years, that is, $t_{E}=3$. We chose the truncation time τ=1.5 years. Furthermore, the maximum follow‐up time per patient was set to τ. We assumed piecewise constant hazards in group C, as detailed in Section 2.3.1, with $t_{0}=0.8$, $\lambda_{0}=0.4$ and $\lambda_{C}=0.7$. The hazard rate $\lambda_{I}$ was set according to $\lambda_{0}$, $\lambda_{C}$ and Δ, as the solution of Equation (A3). To emphasize the difference between the true value of these parameters and the “guesses” used in the sample size calculation at the planning stage of the trial, we refer to these parameters as $t_{0,true}$, $\lambda_{0,true}$, $\lambda_{C,true}$, and $\lambda_{I,true}$ in what follows. By contrast, the guesses will be denoted by $t_{0,guess}$, $\lambda_{0,guess}$, $\lambda_{C,guess}$, and $\lambda_{I,guess}$. The changing point in the piece‐wise exponential survival distributions was assumed to remain the same and perfectly guessed over all scenarios, that is, $t_{0}=t_{0,guess}=t_{0,true}=0.8$. The true censoring rate was given by $r_{cens,true}=0.095$, and the initial guess for the censoring rate at the planning stage was $r_{cens,guess}=0.090$ throughout all scenarios. The RMST difference was assumed to be the minimal clinically relevant difference and was therefore not adapted at the interim analysis in the simulations. Only the guesses for the variances of the RMST estimators in the two treatment groups were adjusted based on the observed data. In addition to the possibility of stopping early for efficacy as described above, we also included a non‐binding futility‐stopping rule. In case the conditional power with the initially planned sample size for the second stage took a value below $CP_{min}=20\%$, we assumed that a rational decision would be to terminate the trial early. This defined the non‐binding futility‐stopping rule. In the simulation study, we assume that the suggestion to stop the trial early for futility at the interim analysis is always followed.

Considered scenarios vary in terms of the difference Δ in RMST, timing $t_{int}$ of the interim analysis, and mis‐specification in survival rate guesses. Scenario S1 is the reference scenario; all others only deviate from S1 by one or a few key features, as listed below. In S1, the interim analysis takes place at $t_{int}=1.8$ and Δ=0.075. We multiply $\lambda_{0,true}$ by a factor $g_{0}$ to obtain $\lambda_{0,guess}$ and $\lambda_{C,true}$ by a factor $g_{C}$ to obtain $\lambda_{C,guess}$. In the reference scenario, $guess_{0}$ refers to mis‐specification factors of $g_{0}=0.8$ and $g_{C}=1.1$.

Scenarios S2 and S3 are the considered scenarios under the null hypothesis, that is, Δ=0.0. S2 has the same mis‐specification rates $g_{0}$ and $g_{c}$ as in S1, and S3 has the optimal guesses, called $guess_{1}$ (i.e., $g_{0}=g_{c}=1$).

Scenarios S4 and S5 make the same assumptions as S1; however, the RMST difference is chosen to equal 0.050 and 0.100, respectively.

Scenarios S6 and S7 serve as a comparison to S1 in terms of the timing of the interim analysis. Again, all assumptions are the same as in S1, but with $t_{int}=2.0$ in S6 and $t_{int}=2.2$ in S7.

Moreover, S8 and S9 complement S1 to provide an evaluation of the impact of the mis‐specification of the survival rates. S8 refers to the case of perfect guesses ($g_{0}=g_{C}=1$, called $guess_{1}$) and S9 assumes $g_{0}=0.5$ and $g_{c}=0.8$, called $guess_{2}$.

Scenario S10 is an additional case that illustrates a case in which the initial sample size was larger than needed. Scenario S10 is similar to Scenario S9, but the “true” and “guessed” rates are interchanged. Therefore, we chose $\lambda_{0,true}=0.2$ and $\lambda_{C,true}=0.56$ as well as $g_{0}=2.0$ and $g_{C}=1.25$ with $\Delta_{0}=\Delta =0.075$ and $t_{int}=1.8$.

**FIGURE 2:**
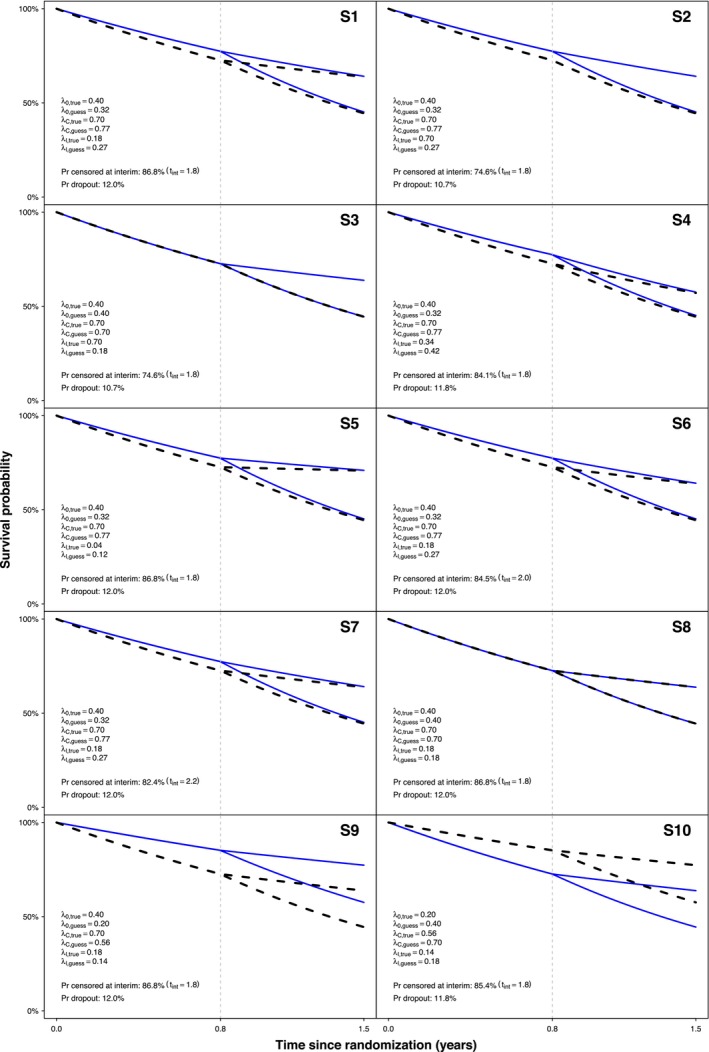
Survival curves based on true survival rates (black) and guessed survival rates (blue) of a random simulation run for S1 to S10 and exemplary data sets. Corresponding censoring probabilities at interim and overall dropout rates are printed for each scenario.

For each scenario, the resulting probability of patients being censored within τ years at the interim analysis, as well as the probability of dropout within τ years (i.e., the probability of being censored within τ years at the final analysis) can be found in Figure 2. This figure also displays the truly generated survival curves (in black) as well as those corresponding to the guessed survival rates (in blue). Note that S1, S6, and S7 differ only with respect to the time of interim analysis; hence, the survival curves are the same.

To facilitate power assessment, in all scenarios under the alternative hypothesis (i.e., when Δ≠0), we assumed that the minimal clinically relevant effect size corresponds to the generated RMST difference (i.e., $\Delta_{0}=\Delta$). For scenarios under the null hypothesis (S2 and S3), we assumed that $\Delta_{0}=0.075$ is chosen to design the trial, as in the reference scenario.

Regarding the interim analysis, we proceeded as follows: Whenever the interim data suggested stopping for efficacy or futility, the trial was stopped early. If the interim data suggested continuing with a second stage, the sample size of the newly recruited patients for the second stage was determined using the conditional power principle. If the conditional power based on the maximally feasible sample size for the second stage was lower than 80%, we continued with this maximally feasible sample size. If the conditional power observed at interim (without including any further patients in the second stage) was already larger than 80%, then no further patients were recruited in the second stage, and the test decision was based on the data from the interim analysis combined with the second‐stage data, the latter consisting only of pipeline data. Otherwise, the second‐stage sample size was calculated as the smallest sample size such that a conditional power of at least 80% could be reached (cf. Section 2.3).

### 3.2 Simulation Results

We evaluated ten scenarios with respect to different performance measures, especially power, sample size, and early stopping probabilities. The results are presented in three tables and additional figures. Table 1 shows the overall evaluation with respect to the ten scenarios with presenting the true treatment effect Δ together with the optimal sample size in a fixed sample size design computed using either the true parameter values ($n_{fix,true}$) or the initial guesses ($n_{fix,guess}$). The number of patients included in the first stage, $n_{1}$, is also provided. Moreover, the table provides the results for the average number of pipeline patients $𝔼[{\bar{n}}_{1}]$, the average stage two sample size $𝔼[n_{2}|cont]$ conditional on continuing the trial, that is, no early stop for futility or efficacy, the overall average sample size $𝔼[n_{total}|cont]$ conditional on a trial continuation, the unconditional overall average sample size $𝔼[n_{total}]$, the overall power $Pow_{RMST}^{\left(1\right)}$ (respectively type‐I error rate $TOER_{RMST}^{\left(1\right)}$ for S2 and S3) according to the truncated data approach (denoted by (1), see below). Additionally, it presents the overall power $Pow_{n_{fix,guess}}$ (respectively type‐I error rate) for a trial with a sample size of $n_{fix,guess}$ and the expected conditional power 𝔼[CP] computed at interim analysis. The probabilities of an early trial stopping at interim analysis for efficacy (${\mathbb{P}}_{eff}$) and futility (${\mathbb{P}}_{fut}$) are also given. For completeness, we also provide first and third quartiles Q1 and Q3 in addition to averages.

**TABLE 1:** Performance evaluation of the RMST‐based test for S1–S10 with $n_{MC}=10\;000$ and L=100. Δ: True difference in RMST; $t_{int}$: Time point of interim analysis; $guess_{0}$ refers to $g_{0}=0.8$ and $g_{c}=1.1$, $guess_{1}$: $g_{0}=1.0$ and $g_{c}=1.0$, $guess_{2}$: $g_{0}=0.5$ and $g_{c}=0.8$, $guess_{3}$: $g_{0}=2.0$ and $g_{c}=1.25$ with $\lambda_{0,true}=0.2$ and $\lambda_{C,true}=0.56$; $n_{fix}$: Total optimal fixed design's sample size for true or guessed parameter assumptions; (*) deviations from 1444 occur due to Monte Carlo simulation error; $n_{1}$: First stage patients; $𝔼[{\bar{n}}_{1}$]: Expected number of pipeline patients at interim analysis (i.e., not observed until τ); $𝔼[n_{2}|cont]$: Expected second‐stage sample size conditional on not stopping early for futility or efficacy; $𝔼[n_{total}|cont]$: Overall expected sample size conditional on not stopping early for futility or efficacy; $𝔼[n_{total}]$: Overall expected sample size together; Q1;Q3: Lower and upper quartile; $Pow_{RMST}^{\left(1\right)}$: Overall power based on truncated data estimation; $TOER_{RMST}^{\left(1\right)}$: Type‐I error rate (only for S2 and S3) based on truncated data estimation; 𝔼[CP]: Conditional power based on observed interim test statistic and updated sample size for second stage; ${\mathbb{P}}_{eff}$: Probability of stopping for efficacy; ${\mathbb{P}}_{fut}$: Probability of stopping for futility. Note that all reported sample sizes refer to both treatment groups together.

Scenario		$n_{fix,true}$	$n_{1}$	$𝔼[{\bar{n}}_{1}]$	$𝔼[n_{2}|cont]$	$𝔼[n_{total}|cont]$	$𝔼[n_{total}]$	$Pow_{RMST}^{\left(1\right)}$	𝔼[CP]	${\mathbb{P}}_{eff}|{\mathbb{P}}_{fut}$
		($n_{fix,guess}$)			(Q1; Q3)	(Q1; Q3)	(Q1; Q3)	or $TOER_{RMST}^{\left(1\right)}$	(Q1; Q3)	in %
								($Pow_{n_{fix,guess}}$) in %	in %	
**S1**	Reference	1444	772	453	540	1312	1268	74.5	78.6	5.4|2.8
	(Δ=0.075;	(1272)			(160;941)	(932;1713)	(854;1663)	(74.9)	(80.0;80.0)	
	$t_{int}=1.8;$									
	$guess_{0})$									
**S2**	Δ=0	1444	772	438	989	1761	1394	2.4	69.4	0.0|37.2
		(1272)			(936;1136)	(1708;1908)	(772;1908)	(2.5)	(60.0; 80.0)	
**S3**	$\Delta =0,guess_{1}$	1440*	874	496	1077	1951	1598	2.3	71.3	0.0|32.8
		(1440*)			(934;1286)	(1808;2160)	(874; 2160)	(2.5)	(63.1; 80.0)	
**S4**	Δ=0.05	3214	1724	1000	1208	2932	2840	73.6	78.7	5.3|2.3
		(2842)			(361;2078)	(2085;3802)	(1911; 3703)	(75.0)	(80.0;80.0)	
**S5**	Δ=0.1	818	438	259	305	743	713	75.1	78.4	7.6|2.5
		(720)			(89;535)	(527;973)	(472;935)	(74.8)	(80.0;80.1)	
**S6**	$t_{int}=2.0$	1444	858	453	473	1331	1257	74.5	78.7	11.8|3.9
		(1272)			(94;858)	(952;1716)	(858;1609)	(74.9)	(80.0;80.0)	
**S7**	$t_{int}=2.2$	1444	944	453	427	1371	1261	75.7	78.8	20.0|5.8
		(1272)			(60;805)	(1004;1749)	(944;1565)	(74.9)	(80.0;80.0)	
**S8**	$guess_{1}$	1440*	874	513	527	1401	1335	76.4	79.4	10.9|1.6
		(1440*)			(127;871)	(1001;1745)	(883; 1666)	(79.9)	(80.0; 80.0)	
**S9**	$guess_{2}$	1444	590	346	546	1136	1091	67.7	75.3	1.3|7.0
		(972)			(261; 868)	(851;1458)	(749; 1458)	(63.3)	(73.0; 80.0)	
**S10**	$guess_{3}$	974	874	578	312	1186	1068	82.9	80.3	37.7|0.2
		(1440*)			(54;480)	(928;1354)	(874; 1169)	(92.6)	(80.0; 80.1)	

Table 2 puts a focus on the different testing options, to compare RMSTs, in terms of how to treat the data of the first and second stage in the testing strategy. The power values are calculated based on the three combination test variants previously described:

(1) referring to the truncated data approach, that is, with second‐stage test statistic given by $Z_{2}$ as defined in Section 2.2.2,
(2) derived from the approach by Desseaux and Porcher [11], that is, with second‐stage test statistic given by $Z_{2}^{\prime }$ as defined in Section 2.2.3,
(3) calculating the effect size estimate based on truncated data and the information estimate according to Desseaux and Porcher [11], that is, with second‐stage test statistic given by $Z_{2}^{\prime \prime }$ as defined in Section 2.2.3.

**TABLE 2:** Overall power respective type‐I error rate (S2 and S3) evaluation with the restricted mean survival time based test (RMST). Results are based on $n_{MC}=10\;000$ and L=100 and are presented in percent. Δ: Difference in RMST; $t_{int}$: Time‐point of interim analysis; $guess_{0}$ refers to $g_{0}=0.8$ and $g_{c}=1.1$, $guess_{1}$: $g_{0}=1.0$ and $g_{c}=1.0$, $guess_{2}$: $g_{0}=0.5$ and $g_{c}=0.8$, $guess_{3}$: $g_{0}=2.0$ and $g_{c}=1.25$ with $\lambda_{0,true}=0.2$ and $\lambda_{C,true}=0.56$; $n_{fix,true}$: Total optimal fixed design's sample size (for minimal clinically relevant effect size $\Delta_{0}=\Delta$ apart from the $H_{0}$ scenarios S2 and S3 where $\Delta_{0}=0.075$ as in the reference scenario); (*) deviations from 1444 occur due to Monte Carlo simulation error; (1): Truncated data estimation, (2): Based on Desseaux & Porcher, (3): Combined approach.

			Power and type‐I error rate in %		
Scenario		$n_{fix,true}$	RMST:(1)	RMST:(2)	RMST:(3)
**S1**	Reference	1444	74.5	77.6	75.4
	(Δ=0.075;				
	$t_{int}=1.8;$				
	$guess_{0})$				
**S2**	Δ=0	1444	2.4	2.5	2.6
**S3**	$\Delta =0,guess_{1}$	1440*	2.3	2.2	2.7
**S4**	Δ=0.05	3214	73.6	77.4	75.0
**S5**	Δ=0.1	818	75.1	77.5	76.1
**S6**	$t_{int}=2.0$	1444	74.5	77.8	75.5
**S7**	$t_{int}=2.2$	1444	75.7	78.1	76.2
**S8**	$guess_{1}$	1440*	76.4	80.1	77.9
**S9**	$guess_{2}$	1444	67.7	69.8	68.3
**S10**	$guess_{3}$	974	82.9	86.9	84.8

Results for the log‐rank test and testing a difference in τ‐year survival are presented in Table A1 in the Appendix, also using the three combination test variants (1), (2), (3), for completeness. Note that these combination tests are based on the same weights as for the RMST‐based tests.

#### 3.2.1 Average Sample Size and Sample Size Increase and Reduction

**FIGURE 3:**
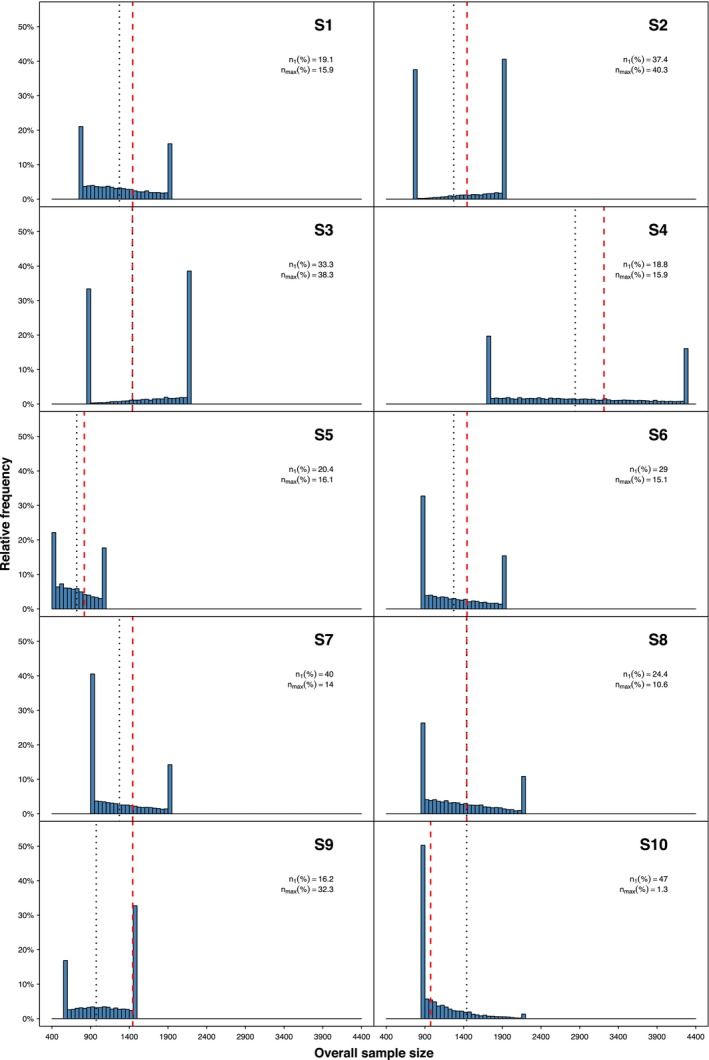
Histograms describing relative frequencies of total sample sizes in S1 to S10 according to the RMST based test ($n_{MC}=10\;000$). Vertical red dashed lines present sample sizes of a fixed sample size design with true parameter values, vertical black dotted lines with initially guessed parameter values. $n_{1}(\%)$: Proportion of $n_{total}=n_{1}$; $n_{max}(\%)$: Proportion of $n_{total}=n_{max}$.

Figure 3 presents histograms for the different scenarios in terms of total sample sizes. For comparison, we added reference lines describing the optimal sample size in a fixed sample size design for the initial parameter guesses ($n_{fix,guess}$, black dotted lines) and true parameter values ($n_{fix,true}$, red dashed lines). Peaks in sample sizes are observed at $n_{1}$ due to stopping early for efficacy, futility, or observing a conditional power larger than 80% at interim, leading to not including new patients in stage 2. Peaks are also observed at $n_{max}$. They correspond to cases where the maximal sample size is reached, and the resulting conditional power is ≤80%. The proportions of total sample sizes being equal to $n_{1}$ or $n_{max}$ are provided with each histogram.

Regarding Table 1, we can note that the $n_{fix,true}$ and $n_{fix,guess}$ values depend on the underlying RMST difference as expected, resulting in approximately $n_{fix,true}=1444$ patients for the scenarios with Δ=0.075. When comparing the average number of pipeline patients ($𝔼[{\bar{n}}_{1}]$) with $n_{1}$, we observe in all scenarios that, on average, more than one half of the stage one patients have not been fully observed at the interim analysis yet. The early efficacy stopping probabilities ${\mathbb{P}}_{eff}$ are rather low apart from scenario S10 (when the initial sample size was larger than needed) or scenario S7 (with a late interim analysis). This was expected, since we applied the O'Brien–Fleming type α‐spending function that spends only a little α at the interim analysis and most of it at the final analysis. We can also see the tendency that the larger the Δ or the later the interim analysis, the larger the probability to stop early for efficacy ${\mathbb{P}}_{eff}$. The expected overall sample sizes $𝔼[n_{total}]$ are in most cases below the fixed sample sizes but with no big savings in sample size (cf. also Figure 3 for a visualization of the observed overall sample sizes). Note that the overall sample sizes can be split into $n_{1}$ and $𝔼[n_{2}]$, where the latter is not presented in its unconditional manner in the table. Only for S10, where the sample size was clearly overestimated at the trial start, the expected overall sample size is clearly larger than $n_{fix,true}$. One can also note that a later interim analysis comes along with a decreased second‐stage sample size due to a larger $n_{1}$. Please be aware of the fact that the fixed design's sample sizes $n_{fix,true}$ are the “perfect” sample sizes, knowing the exact values of all underlying values. The corresponding sample sizes calculated based on the guessed parameter assumptions at the trial start are given by $n_{fix,guess}$ and are more interesting to compare with when assessing the usefulness of sample size recalculation. We also report expected values of the second stage and overall sample size conditional on no early stopping. Those values highlight which sample sizes to expect when the trial does not stop early. They better illustrate the potential benefits of sample size recalculation, as compared to fixed designs. In all cases apart from under the null hypothesis and S10, sample size is still saved on average (compare $𝔼[n_{total}|cont]$ with $n_{fix,true}$).

#### 3.2.2 Power and Type‐I Error Control

The overall power values $Pow_{RMST}^{\left(1\right)}$ (referring to rejecting the null hypothesis either at the interim analysis or at the final analysis according to the truncated data approach (1)) are not precisely hitting 80% in the scenarios under the alternative hypotheses. The slight underpowering in most of the scenarios can be explained by the fact that a maximal sample size limit $n_{max}=1.5\cdot n_{fix,guess}$ was introduced (but no minimal sample size limit above $n_{1}$ to “compensate”). Scenario S10 leads to a slight overpowering and reduces the amount of overpowering compared to that of the initial planning assumptions. This illustrates that sample size recalculation can lead to improvements in terms of sample size and power, but that it cannot fully compensate for guesses that are too far off at initial planning. Under the null hypothesis, the type‐I error rate of 2.5% was maintained in both scenarios S2 and S3 (cf. $TOER_{RMST}^{\left(1\right)}$). Furthermore, the conditional power values are approximately close to 80% whenever Δ≠0. Slight underpowering in some cases can again be explained by the situations where the updated sample size was chosen by the maximally feasible sample size $n_{max}$ when the actually recalculated sample size based on the conditional power exceeded that value. Only the initially overpowered S10 guarantees a conditional power of 80% on average. Moreover, note that with $n_{MC}=10\;000$, the Monte Carlo simulation error is 0.3% (cf. [34]). In almost all cases under the alternative hypothesis, approximately 75% of all the conditional power values take the value 80% (cf. Q1 and Q3). We want to highlight that S8 refers to correct planning assumptions with respect to the survival rates. Here, we observe a small reduction in the overall sample size compared to a fixed design, a probability of 12.5% for an early stop after the first stage, an overall power of 76.4% as well as a conditional power value of 79.4%. Regarding the two corresponding scenarios S9 and S10, we can note the following: With the too optimistic planning assumptions (S9), a power increase is possible but limited by $n_{max}$. With the overly pessimistic planning assumptions (S10), a marginal decrease in sample size of approximately 400 patients on average is possible. However, the power remains slightly overestimated.

#### 3.2.3 Comparison of Different Combination Tests

Table 2 summarizes the power (for Δ≠0) and type‐I error (for Δ=0) results for three different techniques to deal with the left‐truncated stage 2 data (i.e., approaches (1), (2), and (3)). Corresponding results for the log‐rank and τ‐year survival‐based test are presented in Table A1. All initial sample sizes, as well as sample size updates, were calculated to achieve the desired conditional power values for the restricted mean survival time. The two additional tests (log‐rank test and τ‐year survival‐based test) are applied to exactly the same sample sizes and data sets at the interim and final analysis as for the restricted mean survival time‐based test. When considering the RMST‐based power calculations $Pow_{RMST}$ in Table 2, we can note that Approach (2) derived from Desseaux and Porcher addresses our power requirements best. For Δ≠0, the power values are considerably larger than for (1) and (3), and for Δ=0, they do not exceed 2.5%. The combined approach (3) attains higher power values than the truncated approach (1) under Δ≠0, but no approach always assures a power value of 80%. Moreover, the combined approach (3) comes along with larger type‐I error rates. However, those are still within the Monte Carlo error margin. Hence, we can note that the truncated data that we have at the second stage are not negligible, and different approaches for dealing with them result in performance differences.

#### 3.2.4 Comparison With Alternative Tests, Not Specifically Comparing RMSTs

The performance of the log‐rank‐based (LR) and τ‐year (TY) survival‐based test is quite different from the RMST‐based test approach (cf. Tables 2 and A1). In all scenarios (with Δ≠0), the power values of the LR and TY tests are above 90%, often close to 100%. The type‐I error rate performance is within the range of the Monte Carlo error allowance for both tests. The large power values for Δ≠0 can be explained by the underlying data. Since the data structure was inspired by a clinical example that included a delayed treatment response, the difference in survival at τ‐years is the largest compared to everything observed before. This leads to the very large observed power values with that approach. The performance of the LR test aligns with results from the literature [35]. The three different techniques to deal with the left‐truncated second‐stage data do not have a clear influence on the large power values.

#### 3.2.5 Summary of the Simulation Results

To sum up with respect to the four evaluation goals of our simulation study, we can note the following: The new type of clinical trial design is possible. First, type‐I error rate compliance was assured under consideration of the Monte Carlo simulation error for all variants considered. Second, sample size recalculation could indeed mitigate the impact of incorrect initial guesses at the time of trial planning, in terms of suboptimal sample size and power. Sample sizes were often reduced when initially assumed too large and often increased when initially assumed too small. The worse the initial guesses, the larger the benefits of sample size recalculation turned out to be. Third, the RMST‐based approach attained smaller power values than the log‐rank and τ‐year survival approaches, as previously noted in the literature for scenarios with delayed treatment effects. Fourth, the timing of the interim analysis did not substantially affect the power, but only the probability of early termination and the sample size of the second‐stage data. One could expect that the more data available at the interim analysis and the more reliable the conditional power calculation, leads to observed power values closer to the target power. However, this was not observed in our simulation scenarios (when comparing S1, S6, and S7).

## 4 Example of Planning of an Adaptive Trial

In the following, we present a simplified version of our motivating example from a biostatistical consultation. The consultation was about the design of a clinical trial at a university hospital, and we believe that the above methodology could have provided an interesting framework to consider. For confidentiality reasons, some details of this case do not coincide with those presented below (e.g., the expected values of the rates used for sample size calculation). The background was that previous studies suggested that obesity is a major risk factor for atrial fibrillation (AF). Therefore, the idea was to administer some add‐on drug for weight loss with the goal of reducing the risk of AF, among a specific population of patients at high risk of AF. A double‐blind, randomized controlled trial was therefore considered to compare standard treatment (control group) with the standard plus add‐on treatment (intervention group). The envisaged randomization ratio was 1:1. The endpoint was the time until AF diagnosis (or death, whichever comes first, although few or no deaths were expected). AF could be diagnosed at any time during the follow‐up by an implantable cardiac monitor. All patients would receive the implant before randomization (see, e.g., [36] for a similar use of an implantable loop recorder). Recruitment was planned to last 3 years, that is, $t_{E}=3$. The restricted mean survival time was considered an interesting and clinically relevant endpoint at the planning stage for two main reasons. First, non‐proportional hazards were expected. Indeed, it was hypothesized that the add‐on treatment would need time to lead to a clinically relevant weight loss that, in turn, would reduce the risk of AF. Hence, a delayed treatment effect was expected: The survival curves were expected to differ only after some time. Second, because of ethical and logistical considerations, the implantable cardiac monitor could not be kept too long by the patients. It was envisaged to remove them from all patients after 1.5 years. Consequently, all patients were planned to be followed up for the same duration, and no information on the outcome would be available after τ=1.5. Hence, no loss of information would occur when comparing RMSTs instead of comparing hazards. This is different from what happens in most trials, as usually the follow‐up duration varies substantially from one patient to another. At the time of trial planning, there were substantial debates and considerable uncertainty about the rates of AF that should be expected in each arm, resulting in questionable accuracy of the sample size calculation. Logistical and financial considerations pushed towards trial initiation despite this uncertainty, and it was argued that there was no ethical issue preventing trial initiation. Hence, a two‐stage trial with sample size recalculation at interim analysis was considered an interesting option. Censored data caused by dropout were expected. Some patients would ask for the implant to be removed prematurely, for example, if feeling discomfort presumably caused by the implant.

For the assumed effect size, a minimal clinically relevant effect size of $\Delta_{0}=0.05$ could be defined. An interim analysis at $t_{int}=1.8$ years after trial start was deemed logistically feasible. Let us assume $\lambda_{0,guess}=0.4$ in $\left[0;t_{0}=0.8\right)$ and $\lambda_{C,guess}=0.7$ in $\left[0.8;t_{int}+\tau \right)$ for the piece‐wise exponential survival distributions. A treatment effect was not expected before approximately 0.8 years (≈10 months) because the weight loss and related expected benefits would take time to occur. The relevant hazard rate $\lambda_{I,guess}$ for the sample size calculation is chosen such that $\Delta_{0}$ holds true, see Equation (A3). The change time in hazard rate $t_{0}$ is assumed to be approximately correct, and therefore it will not be updated in the sample size recalculation at interim analysis. Additionally, we assume a dropout rate $r_{cens,guess}=r_{cens,true}=0.1$, which could be estimated from former studies using a similar implantable cardiac monitor. This corresponds to expecting approximately 12% of dropout.

An interesting adaptive design to consider could be to assume only efficacy but no futility stopping and plan with variant (2), inspired by Desseaux and Porcher [11] presented earlier, as it performed well in the simulation study. With α=0.025 and β=0.2, the required sample size can now be calculated. According to Equation (8), the total sample size initially computed at trial initiation per group n is 1629 because $\sigma_{1*}=1.013$ (with L=100 and $n_{1}=10\;000$ as in the algorithm presented in Appendix A). Thus, we need to include $n_{1}=n\cdot t_{int}/t_{E}=978$ per group in the first stage, before proceeding to interim analysis and sample size recalculation. The resulting weights for the combination test at the final analysis would be $w_{1}=0.664$ and $w_{2}=0.748$ (cf. Section 2.4).

## 5 Discussion

In this work, we presented three different approaches for dealing with censored data in adaptive trial designs with the option to adapt the sample size together with the restricted mean survival time as the primary endpoint. We compared their performances through an extensive simulation study, and moreover, we compared their performance with the τ‐year survival approach as well as the log‐rank test as alternative testing strategies. The latter was included as it is still frequently applied in the non‐proportional hazards setting, even though it is generally not recommended. Our work was motivated by an example from a biostatistical consultancy for which the suggested method would have been interesting to consider at the planning stage. Note that in this example, the value τ needed for the RMST was given naturally. Often, this is not the case and further efforts are required for determining that value [37]. Our example from the consultancy also served as an illustration of the new method in this article. We used the combination test approach to compare RMST in an adaptive clinical trial. To our knowledge, this approach has not been described before, although it could be relevant beyond the context that motivated this work. We focused on sample size recalculation and the situation where a delayed‐treatment effect is expected at the planning stage of the trial. However, a similar approach could be relevant either in other adaptive trial design settings than only sample size adaptations or in other non‐proportional hazards settings; in the latter broader setting, being probably even more useful, as RMST designs require larger sample sizes in the specific case of delayed responses.

All approaches maintained the type‐I error rate within the range of the Monte Carlo simulation error. The three RMST‐based approaches came along with a slight underpowering. Note that in several scenarios, a power of 80% could not be reached by construction due to the definition of $n_{max}$. However, we have nicely seen that in the case of too optimistic planning, that is, a too small initially planned sample size (cf. S9), an increase in overall power is possible with the new approach. Likewise, if the planning is too pessimistic (cf. S10), that is, a too large initially planned sample size is chosen, a decrease in sample size is possible. Moreover, we have observed quite different power values depending on how the first‐ and second‐stage data were combined. The largest power values could be attained with Approach (2). Therefore, we recommend Approach (2). This approach does not use estimators for left‐truncated data. The modest small sample properties of estimators for left‐truncated data have been noticed before, especially when the risk sets are small for early follow‐up times [38]. This is to some extent the case in our settings, as it might be apparent from Figure 1. This might explain why Approach (2) performs best. On a related note, we observed that an estimator of $\sigma_{21}$ that used the pipeline data $X_{2}^{\left(1\right)}$ only performed very poorly, unlike the estimator defined by Equation (A2) (results not shown). The conditional power values were close to the desired 80% under the alternative, but also here potentially limited from above due to the choice of $n_{max}$. In the histograms, peaks occurred at $n_{1}$ and $n_{max}$ due to the sample size recalculation approach applied. All other values in between were the results from calculations ensuring a specific conditional power value. We wanted to highlight that this pattern is highly dependent on the sample size recalculation approach used. We used a “simple” sample size recalculation approach to illustrate the general approach and facilitate power assessment. However, alternative sample size recalculation approaches might be better suited in specific contexts, and they could perform very differently. For instance, a recalculation resulting from a “promising zone” approach [39] would perform very differently, as the second‐stage sample size is always at least the size of the initially planned one. It would lead to much higher power results, but at the cost of larger average sample sizes.

Power values for the RMST‐based approach were smaller than for the test comparing τ‐year survival probabilities and the log‐rank test, applied to the same sample sizes. Also, for these tests, the type‐I error rates were maintained within the Monte Carlo error margins. Power values for the scenarios under the alternative hypothesis were all above 90% for the τ‐year survival approach and log‐rank test. Such a power difference compared to the RMST‐based approach is also known from the literature [35]. This serves as a good reminder that, when planning a trial, one has to balance the choice of the best estimand and accompanying test in terms of interpretation and robustness against statistical power. In this work, we focus on the delayed treatment effect setting where all patients are followed up for the same duration. Other non‐proportional hazard settings would likely lead to different conclusions when comparing the powers of different tests for different estimands, see, for example, Maggir et al. [35] and references therein. In the delayed treatment effect setting that we considered, if $t_{0}$ happens to be very early, we are close to a proportional hazards setting. In that case, using a log‐rank test and comparing hazard ratios is very appealing. If $t_{0}$ is close to τ, comparing τ‐year survival probabilities is very appealing. The approach consisting of comparing RMSTs has probably its place when one expects a situation between these two extremes.

Moreover, we want to highlight that we consider the new research results presented here rather as a framework than an entirely final “out‐of‐the‐shelf‐solution”. This means that one might want to choose different parameter values from those we used, for example, to target a conditional power value of 90% instead of 80% or use lower or upper limits of some confidence intervals instead of point estimates for the rates (to be conservative), when computing the conditional power.

In general, sample size adaptations can be thought of in different ways when considering time‐to‐event data: One can either prolong the accrual time or change the accrual rate, where the physician's decision on feasibility is important to keep in mind. In this work, we only considered one value of τ due to the clinical example in mind. However, the RMST depends strongly on the choice of that value, and we recommend considering several values and their consequences in other application scenarios. Also, in the simulations, we chose to perform a relatively small number of simulations (L=100) to estimate the key parameters needed for computing the conditional power (to save computational time). Unshown results for a few runs using L=1000 instead were very similar. However, in practice, a larger number should be used to gain precision and increase reproducibility.

There are several ways in which our suggested methods could be extended in future work. We only adapted the nuisance parameters with the methods we provide, but the underlying effect size guess could also be adapted. Another extension could be to use the predictive power as a possible alternative to the conditional power, even though it is also not considered as generally favorable [40]. Considering other parametric survival distributions for the sample size recalculation, for example, Weibull or other piecewise constant hazard situations, could also be worth considering. The latter would be particularly interesting for dealing with any non‐proportional hazard setting of interest. Alternatively, one could compute Kaplan–Meier curves estimated under the constraint $\Delta =\Delta_{0}$, as in Reference [41], instead of using parametric distributions and constrained maximum likelihood estimation as in Appendix A. However, we hypothesize that the limited sample size available at interim analysis will lead to a bias‐variance tradeoff favoring a parametric approach. Finally, shrinkage or Bayesian approaches to update the guesses at the interim analysis could be interesting to consider. They might be a good compromise between using the original guesses (as in a fixed design) and letting the interim data of a limited sample size speak freely (as we considered in this work). This would likely substantially reduce the variability in the second‐stage sample size, which might be important in some contexts.

For addressing guidelines by the authorities, an approach allowing for covariate adjustment would also be appealing to consider. However, Bauer and Posch [42] have nicely pointed out the challenges it implies: For mid‐trial sample size adaptations, only the information provided by patients having obtained an event or who were censored before the interim analysis can be used as a source of information, but not additional patient characteristics. Otherwise, it can lead to increased type‐I error.

## 6 Conclusion

We showed how to plan and analyze an adaptive trial with the restricted mean survival time as the primary endpoint. In particular, we provided the computational details for performing sample size recalculation at interim analysis, in the context of delayed treatment effect. This provides new tools to plan trials in situations when non‐proportional hazards are expected, and a large uncertainty about key parameters for sample size calculation is prevalent. This work also opens up possibilities for different types of adaptations with the RMST as the primary endpoint. The t‐year approach may be better to apply in terms of power and sample size when facing delayed treatment effects. The RMST seems especially appealing when not knowing what to expect (e.g., doubt about delayed treatment effect or diminishing effect in the non‐proportional hazards setting, cf., e.g., Figure 1 in Reference [32] for illustrations) or in situations with diminishing effects (e.g., [32]).

## Funding

The authors have nothing to report.

## Conflicts of Interest

The authors declare no conflicts of interest.

## Data Availability

The data that support the findings of this study are available in the Supporting Information of this article.

## Appendix A

### Definition Based on Counting Processes

As mentioned in Section 2.2.2, as $n_{1}\to \infty$, we have $\sqrt{n_{1}}\{\hat{\Delta }(X_{1})-\Delta \}∼N(0,\sigma_{1}^{2})$, for some $\sigma_{1}>0$, and $\sqrt{n_{2}+{\bar{n}}_{1}}\{\hat{\Delta }(X_{2})-\Delta \}∼N(0,\sigma_{2}^{2})$, for some $\sigma_{2}>0$, as $n_{2}\to \infty$ and ${\bar{n}}_{1}\to \infty$, see, for example, Example IV.3.8 in [25]. Note that ${\bar{n}}_{1}\to \infty$ is equivalent to $n_{1}\to \infty$, assuming that the probability of observing stage 1 patients for whom further data collection after the interim analysis is not zero.

If there was no dropout nor interim analysis, $\sigma_{1}^{2}$ would simply be $(\xi_{I}^{2}+\xi_{C}^{2})/2$ with $\xi_{i}$ being the standard deviation of $X_{\tau }=\min(T,\tau )$ in group i∈{I,C}. However, because of dropout and additional censoring because of the interim analysis, $\sigma_{1}^{2}$ will be larger. As pointed out in, for example, Example IV.3.8 in [25] or [43], the variance of the RMST estimator in each group i∈{I,C}, using data $X_{k}$, for k∈{1,2}, can be computed as

(A1)
$${\left\{{\hat{\sigma }}_{{\hat{\mu }}_{\tau }}^{\left[i\right]}(X_{k})\right\}}^{2}=\int_{0}^{\tau }{\left(\int_{t}^{\tau }{\hat{S}}_{X_{k}}^{\left[i\right]}(x)dx\right)}^{2}\frac{dN_{X_{k}}^{\left[i\right]}(t)}{{\left\{Y_{X_{k}}^{\left[i\right]}(t)\right\}}^{2}},$$

where $N_{X_{k}}^{\left[i\right]}(t)$ counts the number of observed events before t and $Y_{X_{k}}^{\left[i\right]}(t)$ counts the number of subjects observed at risk at t− (i.e., “just” before t), in treatment group i∈{I,C}. That is, with stage 1 data $X_{1}$, we use $N_{X_{1}}^{\left[i\right]}(t)=\sum_{j=1}^{n_{1}}1({\tilde{T}}_{j}^{\left(1\right)}\leq t,A_{j}=i)\delta_{j}^{\left(1\right)}$ and $Y_{X_{1}}^{\left[i\right]}(t)=\sum_{j=1}^{n_{1}}1({\tilde{T}}_{j}^{\left(1\right)}\geq t,A_{j}=i)$. With stage 2 data $X_{2}$, we use $N_{X_{2}}^{\left[i\right]}(t)=\sum_{j=n_{1}-{\bar{n}}_{1}+1}^{n_{1}+n_{2}}1(L_{j}<t,{\tilde{T}}_{j}\leq t,A_{j}=i)\delta_{j}$ and $Y_{X_{2}}^{\left[i\right]}(t)=\sum_{j=n_{1}-{\bar{n}}_{1}+1}^{n_{1}+n_{2}}1(L_{j}<t\leq {\tilde{T}}_{j},A_{j}=i)$. With these notations, the Kaplan–Meier estimator of the survival function at time s is given by ${\hat{S}}_{X_{k}}^{\left[i\right]}(t)=\prod_{s\leq t}\left\{1-dN_{X_{k}}^{\left[i\right]}(s)/Y_{X_{k}}^{\left[i\right]}(s)\right\}$, and that of the restricted mean survival time by ${\hat{\mu }}_{\tau }^{\left[i\right]}(X_{k})=\int_{0}^{\tau }{\hat{S}}_{X_{k}}^{\left[i\right]}(t)dt$, for each group i∈{I,C}, which leads to $\hat{\Delta }(X_{k})=\int_{0}^{\tau }\left({\hat{S}}_{X_{k}}^{\left[I\right]}(t)-{\hat{S}}_{X_{k}}^{\left[C\right]}(t)\right)dt$. Consistent estimators of $\sigma_{1}$ and $\sigma_{2}$ can therefore be obtained by

$$\begin{array}{ll} {\hat{\sigma }}_{1}^{2} & =n_{1}/2\left[{\left\{{\hat{\sigma }}_{{\hat{\mu }}_{\tau }}^{\left[I\right]}(X_{1})\right\}}^{2}+{\left\{{\hat{\sigma }}_{{\hat{\mu }}_{\tau }}^{\left([C\right]}(X_{1})\right\}}^{2}\right],\\ {\hat{\sigma }}_{2}^{2} & =(n_{2}+{\bar{n}}_{1})/2\left[{\left\{{\hat{\sigma }}_{{\hat{\mu }}_{\tau }}^{\left[I\right]}(X_{2})\right\}}^{2}+{\left\{{\hat{\sigma }}_{{\hat{\mu }}_{\tau }}^{\left[C\right]}(X_{2})\right\}}^{2}\right]. \end{array}$$

Note that ${\hat{\sigma }}_{{\hat{\mu }}_{\tau }}^{\left[i\right]}(X_{k})$, which is the standard error of the RMST estimator ${\hat{\mu }}_{\tau }^{\left[i\right]}(X_{k})$, can be easily computed by the survival package in R, which deals with right‐censored and left‐truncated data. It is also implemented in the survRM2 package in R, however for right‐censored data only, not left truncated data. Two further remarks are the following. First, as usual, $n_{1}/2$ can be replaced by $1/\{(1/n_{1,C})+(1/n_{1,I})\}$, where $n_{1,i}$ is the number of subjects, among the $n_{1}$ observed at interim analysis, which have been randomized to group i∈{I,C}. A similar remark applies to $(n_{2}+{\bar{n}}_{1})/2$. This should lead to better small‐sample‐size properties. Second, in Equation (A1), the denominator ${\left\{Y_{X_{k}}^{\left[i\right]}(t)\right\}}^{2}$ can be replaced by $Y_{X_{k}}^{\left[i\right]}(t)\{Y_{X_{k}}^{\left[i\right]}(t)-dN_{X_{k}}^{\left[i\right]}(t)\}$, to lead to a Greenwood‐type formula (see, e.g., p. 59 in  [20]). Although asymptotically equivalent, this might also lead to better sample size properties, and this corresponds to what is implemented in most software (e.g., in survival and survRM2 packages of R).

### Estimating $\sigma_{1*}$ and $\sigma_{21}$ via Simulations

The asymptotic variance terms $\sigma_{1*}^{2}$ and $\sigma_{21}^{2}$ can be estimated by simulations as follows. Important additional technical details for Step 1(a) below are provided in Sections 2.3.1, Simulating Data in the Delayed Treatment Effect Context: Further Details, and A.

1. For l=1,…,L and $n_{1}$ large:
  (a) Simulate data $\left\{({\tilde{T}}_{j},{\tilde{T}}_{j}^{\left(1\right)},\delta_{j},\delta_{j}^{\left(1\right)},A_{j}),j=1,\ldots ,n_{1}\right\}$. Define $D_{1*}^{\left(l\right)}=\{({\tilde{T}}_{j},\delta_{j},A_{j}),j=1,\ldots ,n_{1}\}$ and $D_{1}^{\left(l\right)}=\{({\tilde{T}}_{j}^{\left(1\right)},\delta_{j}^{\left(1\right)},A_{j}),i=1,\ldots ,n_{1}\}$, using a relevant data‐generating mechanism that reflects what is observed via data $X_{1}$. This means that we compute a large data set that mimics the data observed in $X_{1}$, assuming that an interim analysis did not occur (for $D_{1*}^{\left(l\right)}$) and that it is occurring (for $D_{1}^{\left(l\right)}$).
  (b) Compute
    $$\begin{array}{ll} {\left\{{\hat{\sigma }}_{1*}^{\left(l\right)}\right\}}^{2} & =n_{1}/2\left[{\left\{{\hat{\sigma }}_{{\hat{\mu }}_{\tau }}^{\left[I\right]}(D_{1*}^{\left(l\right)})\right\}}^{2}+{\left\{{\hat{\sigma }}_{{\hat{\mu }}_{\tau }}^{\left[C\right]}(D_{1*}^{\left(l\right)})\right\}}^{2}\right], \end{array}$$
    where ${\hat{\sigma }}_{{\hat{\mu }}_{\tau }}^{\left(i\right)}(D_{1*}^{\left(l\right)})$ is the standard error of the RMST estimator in each treatment group i∈{I,C}, computed from data $D_{1*}^{\left(l\right)}$. That is, as described in Equation (A1), using data $D_{1*}^{\left(l\right)}$ instead of data $X_{1}$. Similarly, use data $D_{1}^{\left(l\right)}$ to compute
    $$\begin{array}{ll} {\left\{{\hat{\sigma }}_{1}^{\left(l\right)}\right\}}^{2} & =n_{1}/2\left[{\left\{{\hat{\sigma }}_{{\hat{\mu }}_{\tau }}^{\left[I\right]}(D_{1}^{\left(l\right)})\right\}}^{2}+{\left\{{\hat{\sigma }}_{{\hat{\mu }}_{\tau }}^{\left[C\right]}(D_{1}^{\left(l\right)})\right\}}^{2}\right]. \end{array}$$
  (c) Compute ${\left\{{\hat{\sigma }}_{21}^{\left(l\right)}\right\}}^{2}$ from the previously computed ${\left\{{\hat{\sigma }}_{1}^{\left(l\right)}\right\}}^{2}$ and ${\left\{{\hat{\sigma }}_{1*}^{\left(l\right)}\right\}}^{2}$ and sample sizes $n_{1}$ and ${\bar{n}}_{1}^{\left(l\right)}=n_{1}-\sum_{j=1}^{n_{1}}1({\tilde{T}}_{j}^{\left(1\right)}<{\tilde{T}}_{j})$, using
    (A2)
    $$\begin{array}{l} \frac{n_{1}}{{\left\{{\hat{\sigma }}_{1*}^{\left(l\right)}\right\}}^{2}}=\frac{{\bar{n}}_{1}^{\left(l\right)}}{{\left\{{\hat{\sigma }}_{21}^{\left(l\right)}\right\}}^{2}}+\frac{n_{1}}{{\left\{{\hat{\sigma }}_{1}^{\left(l\right)}\right\}}^{2}}. \end{array}$$
2. Compute $\sigma_{1*}$, $\sigma_{21}$ and $\sigma_{1}$ as the mean of the L values of ${\hat{\sigma }}_{1*}^{\left(l\right)}$, ${\hat{\sigma }}_{21}^{\left(l\right)}$ and ${\hat{\sigma }}_{1}^{\left(l\right)}$, respectively.

In short, the above Equation (A2) corresponds to ${\hat{I}}_{all}={\hat{I}}_{1}+{\hat{I}}_{2}+o_{p}(n)$ already met in Section 2.2.3, when $n_{2}=0$. It reflects that the total information provided by the patients recruited within the first stage can be decomposed into the sum of the information obtained from their follow‐up data observed before the interim analysis, plus the information obtained from their remaining follow‐up data observed after the interim analysis. This corresponds to the so‐called independent increment covariance structure proved by [21], in the special case where the interim analysis occurs after all patients have been accrued.

### Simulating Data in the Delayed Treatment Effect Context: Further Details

One can simulate data $\left\{({\tilde{T}}_{j},{\tilde{T}}_{j}^{\left(1\right)},\delta_{j},\delta_{j}^{\left(1\right)},A_{j}),j=1,\ldots ,n_{1}\right\}$ mentioned in Step 1(a) of the previous sub‐section as follows. For $j=1,\ldots ,n_{1}$:

1. Generate $U_{j},V_{j},W_{j},B_{j}$ i.i.d. from a uniform distribution on [0,1].
2. Compute $T_{j}^{0}=-\log(U_{j})/\lambda_{0}$.
3. If $j\leq n_{1}/2$, set $A_{j}=C$ and $\lambda_{i}=\lambda_{C}$. If $j>n_{1}/2$, set $A_{j}=I$ and $\lambda_{i}=\lambda_{I}$. Then compute
  $$T_{j}^{i}=-\log(V_{j})/\lambda_{i}\;\text{and}\;T_{j}=\min(\overset{0}{\underset{j}{T}},t_{0})+T_{j}^{i}\cdot 1(T_{j}^{0}>t_{0}).$$
4. Compute $C_{j}=-\log(W_{j})/c$.
5. Compute ${\tilde{T}}_{j}=\min(T_{j},C_{j},\tau )$ and $\delta_{j}=1(T_{j}\leq \min(C_{j},\tau ))$.
6. Generate the entry time $E_{j}$ from a uniform distribution on $\left[0,t_{int}\right]$ and compute $L_{j}=t_{int}-E_{j}$.
7. Compute ${\tilde{T}}_{j}^{\left(1\right)}=\min({\tilde{T}}_{j},L_{j})$ and $\delta_{j}^{\left(1\right)}=1(T_{j}\leq \min(C_{j},\tau ,L_{j}))$.

### Simulating Data Alike Those Observed at Interim Analysis via Constrained Maximum Likelihood Estimation

We assume that initial values of the rate parameters c, $\lambda_{0}$, $\lambda_{C}$ and $\lambda_{I}$ have been used to simulate data and derive the sample size $n_{1}$ of the first stage data as well as to choose appropriate weights $w_{1}$ and $w_{2}$ accordingly. See Section 2.4 for further details about how this can be done.

At the interim analysis, the observed data $X_{1}$ can be used to estimate the parameters c, $\lambda_{0}$, $\lambda_{C}$ and $\lambda_{I}$. These values can in turn be used to simulate data, derive estimates of $\sigma_{21}$ and $\sigma_{1*}$ as described in the sub‐section above and further to derive the second‐stage sample size $n_{2}$ by solving the above Equation (6). This is precisely the data adaptive method and corresponding adaptive trial design that we consider in this manuscript.

To estimate c, $\lambda_{0}$, $\lambda_{C}$ and $\lambda_{I}$ using data $X_{1}$, it seems natural to use maximum likelihood methods. However, we have to keep in mind that we want to simulate data under the specific alternative hypothesis $\Delta =\Delta_{0}$, for which we want to power the trial. Hence, we suggest proceeding via constrained maximum likelihood estimation. The constraint is needed since our piece‐wise constant hazard assumptions imply

(A3)
$$\begin{array}{l} \Delta_{0}+\frac{e^{-\lambda_{0}t_{0}}}{\lambda_{C}}\left(1-e^{-\lambda_{C}(\tau -t_{0})}\right)-\frac{e^{-\lambda_{0}t_{0}}}{\lambda_{I}}\left(1-e^{-\lambda_{I}(\tau -t_{0})}\right)=0, \end{array}$$

which follows from simple algebra detailed in, for example, Appendix of [17]. Hence, $\lambda_{I}$ is a function of $\lambda_{0}$, $\lambda_{C}$, $\Delta_{0}$, τ and $t_{0}$ and it cannot be maximized independently of $\lambda_{0}$, $\lambda_{C}$, for given values of $t_{0}$, τ and $\Delta_{0}$.

Formally, we suggest to estimate the nuisance parameters c, $\lambda_{0}$, $\lambda_{C}$ and $\lambda_{I}$ as the values that maximize the log‐likelihood of the data $X_{1}$, denoted by $\log\{ℒ(X_{1})\}$, under the constraint of Equation (A3). Interestingly, the log‐likelihood can be decomposed as

(A4)
$$\begin{array}{l} \log\{ℒ(X_{1})\}=l_{0}(\lambda_{0})+l_{I}(\lambda_{I})+l_{C}(\lambda_{C})+l_{c}(c), \end{array}$$

where

(A5)
$$\begin{array}{ll} l_{0}(\lambda_{0}) & =\overset{n_{1}}{\underset{j=1}{\sum }}\left\{\delta_{j}^{\left(1\right)}\log(\lambda_{0})1({\tilde{T}}_{j}^{\left(1\right)}\leq t_{0})-\lambda_{0}\min\left(\overset{\left(1\right)}{\underset{j}{\tilde{T}}},t_{0}\right)\right\} \end{array}$$

(A6)
$$\begin{array}{ll} l_{i}(\lambda_{i}) & =\overset{n_{1}}{\underset{j=1}{\sum }}\left\{\delta_{j}^{\left(1\right)}\log(\lambda_{i})-\lambda_{i}\left({\tilde{T}}_{j}^{\left(1\right)}-t_{0}\right)\right\}1({\tilde{T}}_{j}^{\left(1\right)}>t_{0})1(A_{j}=i)\;\text{for}\;i\in \{I,C\} \end{array}$$

(A7)
$$\begin{array}{ll} \text{and}\;l_{c}(c) & =\overset{n_{1}}{\underset{j=1}{\sum }}\left\{1({\tilde{T}}_{j}^{\left(1\right)}\leq L_{j})(1-\delta_{j}^{\left(1\right)})\log(c)-c{\tilde{T}}_{j}^{\left(1\right)}\right\}. \end{array}$$

First, we note that finding c that maximizes Equation (A7) or (A4) is equivalent, and this leads to $ĉ=\{\sum_{j=1}^{n_{1}}1({\tilde{T}}_{j}^{\left(1\right)}\leq L_{j})(1-\delta_{j}^{\left(1\right)})\}/\{\sum_{j=1}^{n_{1}}{\tilde{T}}_{j}^{\left(1\right)}\}$, that is, the usual incidence rate (number of dropouts divided by the total number of person‐time at risk of dropout). We can also note that because of the constraint (A3), $\lambda_{I}$ can be written as a function of $\lambda_{0}$ and $\lambda_{C}$. Hence, informally we can rewrite $l_{I}(\lambda_{I})$ as $l_{I}(\lambda_{0},\lambda_{C})$ and exploit that

$$\begin{array}{ll} & \underset{(\lambda_{I},\lambda_{C},\lambda_{0},c)\in {\left\{{\mathbb{R}}^{+}\right\}}^{4}\;:\;\Delta =\Delta_{0}}{\max}\left\{l_{0}(\lambda_{0})+l_{I}(\lambda_{I})+l_{C}(\lambda_{C})+l_{c}(c)\right\}\\ & \;=\underset{\lambda_{0}\in {\mathbb{R}}^{+}\;:\;\Delta =\Delta_{0}}{\max}\left\{l_{0}(\lambda_{0})+\underset{\lambda_{C}\in {\mathbb{R}}^{+}\;:\;\Delta =\Delta_{0}}{\max}\left[l_{I}(\lambda_{0},\lambda_{C})\right.\right.\\ & \;\left.\left.+\;l_{C}(\lambda_{C})\right]\right\}+\underset{c\in {\mathbb{R}}^{+}}{\max}l_{c}(c). \end{array}$$

The maximization under constraint can therefore be done by two nested calls to a simple optimization function (e.g., optimize() in R).

Note that we could additionally aim to estimate $t_{0}$ from the data $X_{1}$ and update the initial value guessed for this parameter accordingly, when simulating the data and deriving the conditional power and sample size $n_{2}$. Although this is possible in theory, we believe, however, that typical sample sizes $n_{1}$ available at the interim will not be sufficient to do this well. We therefore do not further consider this idea in this manuscript.

**TABLE A1:** Overall power respective type‐I error rate (S2 and S3) evaluation for the log‐rank test (LR) and τ‐year survival based test (TY). Results are based on $n_{MC}=10\;000$ and L=100 and are presented in percent. Δ: Difference in RMST; $t_{int}$: Time‐point of interim analysis; $guess_{0}$ refers to $g_{0}=0.8$ and $g_{c}=1.1$, $guess_{1}$: $g_{0}=1.0$ and $g_{c}=1.0$, $guess_{2}$: $g_{0}=0.5$ and $g_{c}=0.8$, $guess_{3}$: $g_{0}=2.0$ and $g_{c}=1.25$ with $\lambda_{0,true}=0.2$ and $\lambda_{C,true}=0.56$; $n_{fix,true}$: Total optimal fixed design's sample size (for minimal clinically relevant effect size $\Delta_{0}=\Delta$ apart from the $H_{0}$ scenarios S2 and S3 where $\Delta_{0}=0.075$ as in the reference scenario); (*) deviations from 1444 occur due to Monte Carlo simulation error; (1): Truncated data estimation, (2): Based on Desseaux & Porcher, (3): Combined approach.

			Power and type‐I error rate in %					
Scenario		$n_{fix,true}$	LR:(1)	LR:(2)	LR:(3)	TY:(1)	TY:(2)	TY:(3)
**S1**	Reference	1444	97.0	97.0	97.0	97.1	97.1	97.1
	(Δ=0.075;							
	$t_{int}=1.8;$							
	$guess_{0})$							
**S2**	Δ=0	1444	2.3	2.4	2.3	2.4	2.5	2.6
**S3**	$\Delta =0,guess_{1}$	1440*	2.3	2.3	2.3	2.4	2.8	2.7
**S4**	Δ=0.05	3214	97.7	97.7	97.7	97.4	97.4	97.4
**S5**	Δ=0.1	818	97.3	97.3	97.3	97.6	97.6	97.6
**S6**	$t_{int}=2.0$	1444	96.1	96.1	96.1	96.6	96.6	96.6
**S7**	$t_{int}=2.2$	1444	94.2	94.2	94.2	96.7	96.7	96.7
**S8**	$guess_{1}$	1440*	98.4	98.4	98.4	98.4	98.4	98.4
**S9**	$guess_{2}$	1444	92.9	92.9	92.9	92.9	93.0	92.8
**S10**	$guess_{3}$	974	99.8	99.8	99.8	99.7	99.7	99.7
